# The bromodomain and extra-terminal inhibitor CPI203 enhances the antiproliferative effects of rapamycin on human neuroendocrine tumors

**DOI:** 10.1038/cddis.2014.396

**Published:** 2014-10-09

**Authors:** C Wong, S V Laddha, L Tang, E Vosburgh, A J Levine, E Normant, P Sandy, C R Harris, C S Chan, E Y Xu

**Affiliations:** 1 Raymond and Beverly Sackler Foundation Laboratory, 195 Little Albany Street, New Brunswick, NJ 08901, USA; 2 Rutgers Cancer Institute of New Jersey, Robert Wood Johnson Medical School, Rutgers, The State University of New Jersey, 195 Little Albany Street, New Brunswick, NJ 08901, USA; 3 Department of Medicine, Robert Wood Johnson Medical School, Rutgers, The State University of New Jersey, 195 Little Albany Street, New Brunswick, NJ 08901, USA; 4 Center for Systems Biology, Robert Wood Johnson Medical School, Rutgers, The State University of New Jersey, 195 Little Albany Street, New Brunswick, NJ 08901, USA; 5 Department of Pathology, Memorial Sloan-Kettering Cancer Center, New York, NY 10065, USA; 6 Department of Pediatrics, Robert Wood Johnson Medical School, Rutgers, The State University of New Jersey, 195 Little Albany Street, New Brunswick, NJ 08901, USA; 7 School of Natural Sciences, Institute for Advanced Study, 1 Einstein Drive, Princeton, NJ 08540, USA; 8 Constellation Pharmaceuticals, Cambridge, MA 02142, USA

## Abstract

Endogenous c-MYC (MYC) has been reported to be a potential pharmacological target to trigger ubiquitous tumor regression of pancreatic neuroendocrine tumors (PanNETs) and lung tumors. Recently inhibitors of bromodomain and extra-terminal (BET) family proteins have shown antitumor effects through the suppression of MYC in leukemia and lymphoma. In this paper, we investigated the antitumor activity of a BET protein bromodomain inhibitor (BETi) CPI203 as a single agent and in combination with rapamycin in human PanNETs. We found that exposure of human PanNET cell lines to CPI203 led to downregulation of MYC expression, G1 cell cycle arrest and nearly complete inhibition of cell proliferation. In addition, overexpression of MYC suppressed the growth inhibition caused by CPI203 and knockdown of MYC phenocopied the effects of CPI203 treatment. These findings indicate that suppression of MYC contributed to the antiproliferative effects of BETi inhibition in human PanNET cells. Importantly, CPI203 treatment enhanced the antitumor effects of rapamycin in PanNET cells grown in monolayer and in three-dimensional cell cultures, as well as in a human PanNET xenograft model *in vivo*. Furthermore, the combination treatment attenuated rapamycin-induced AKT activation, a major limitation of rapamycin therapy. Collectively, our data suggest that targeting MYC with a BETi may increase the therapeutic benefits of rapalogs in human PanNET patients. This provides a novel clinical strategy for PanNETs, and possibly for other tumors as well.

Human pancreatic neuroendocrine tumors (PanNETs) are a subset of neuroendocrine tumors (NETs) that arise in the islet cells of the pancreas, and are also referred to as islet cell tumors. PanNETs are the second most common pancreatic malignancy, with limited treatment options for unresectable tumors.^1^ Recently, two agents, everolimus and sunitinib, were approved by the US Food and Drug Administration (FDA) for the treatment of unresectable PanNETs^2,3^ based on progression-free survival benefits in PanNET patients.^4,5^ Everolimus, a rapamycin analog (rapalog) and a mammalian target of rapamycin (mTOR) inhibitor, showed antitumor effects in PanNETs. However, even partial response by Response Evaluation Criteria in Solid Tumors (RECIST) were obtained in less than 10% of treated patients.^1^ Therefore, it has been speculated that rapalogs may achieve better antitumor activity in combination with other anticancer agents.

c-MYC (MYC) is a potent oncogene that is frequently deregulated in a variety of cancers. As a transcription factor (TF), it plays a role in many important intracellular programs such as cell proliferation, cell cycle progression, differentiation and apoptosis.^6^ Although deregulation of MYC in PanNETs is ill-defined, Sodir *et al.*^7^ showed that endogenous MYC plays a role in maintaining PanNETs and their microenvironment. By introducing a controllable dominant-negative MYC inhibitor *Omomyc* gene into a *simian virus 40* (SV40)-driven PanNET mouse, the authors demonstrated that inhibition of endogenous MYC triggered regression of tumors, suggesting that targeting MYC may have a clinical potential for human PanNET patients.

Until recently, MYC has been considered ‘undruggable' because there are no ligand-binding pockets in the basic helix-loop-helix leucine zipper domain of the MYC protein. *MYC* gene is regulated by BRD4, a bromodomain and extra-terminal (BET) protein.^8^ There are four proteins in this family - BRD2, BRD3, BRD4 and BRDT. The BET proteins share a common structure with two N-terminal bromodomains that exhibit high levels of sequence conservation as well as an extra-terminal (ET) domain and a more divergent C-terminal recruitment domain. They function at the interface between chromatin remodeling and transcriptional regulation through binding to acetylated lysines on chromatin.^9^ Miyoshi *et al.*^10^ first described a thienodiazepine analog that competitively binds to the acetyl-binding pockets of the BET family protein, resulting in their release from chromatin. CPI203 is a thienodiazepine derivative^11^ that decreased *Myc* mRNA and reduced leukemia burden in a T-cell acute lymphoblastic leukemia mouse model.^12^ Extensive studies of the related small molecule (+)−JQ1 in leukemia and lymphoma have shown that this BET protein bromodomain inhibitor (BETi) achieved antitumor activity through suppression of MYC.^13,14^ The ability of BETi to reduce expression of MYC highlights the promise of this therapeutic strategy to target MYC.

Here, we investigated the antitumor activity of CPI203 as a single agent and in combination with rapamycin in human PanNET cells. CPI203 treatment caused downregulation of MYC and nearly complete growth inhibition in PanNET cells *in vitro* and *in vivo*. Furthermore, combination treatment of CPI203 with rapamycin showed stronger antiproliferative effects and decreased AKT activation in human PanNETs. Taken together, treatment with BETi and rapamycin critically lowered MYC and phospho-AKT, implicating that co-treatment may increase the response rate of patients.

## Results

### Human PanNET cell lines are sensitive to BETi

**Figure 1:**
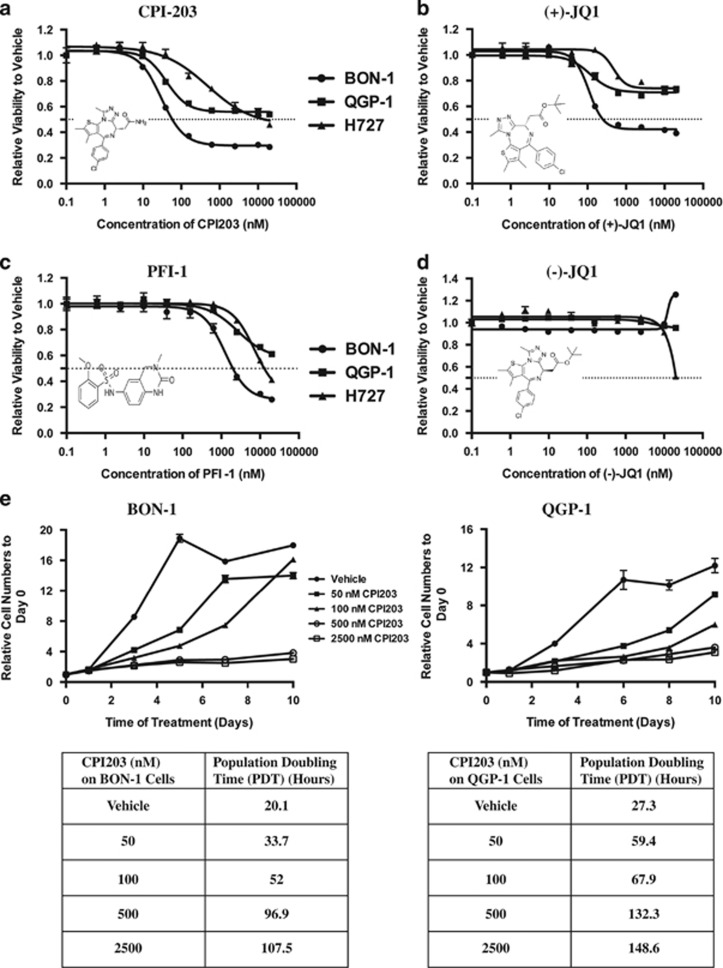

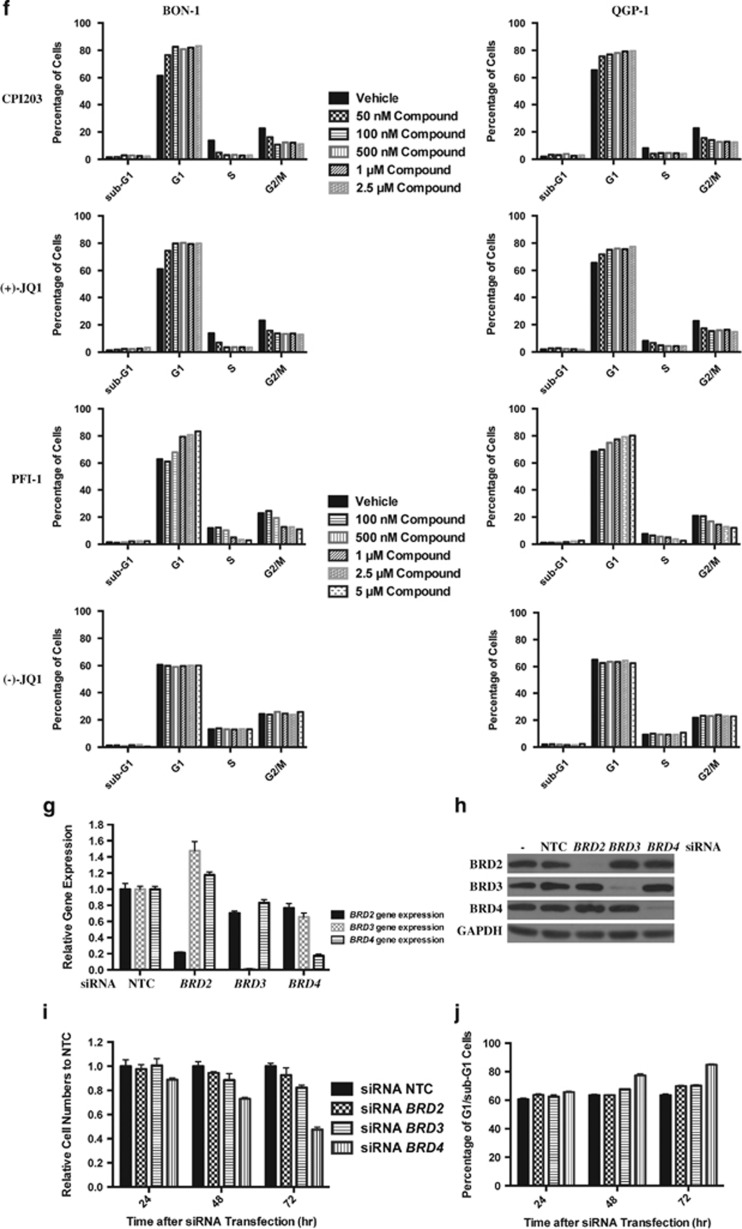
Human PanNET cell lines are sensitive to BETi. (**a**–**d**) Dose-response curves of NET cell lines treated for 72 h with DMSO or CPI203 (**a**), (+)-JQ1 (**b**), PFI-1 (**c**) or (−)-JQ1 (**d**) in nine-point four-fold dilutions starting at 20 *μ*M. Error bars represent S.E.M., *n*=3. Inset: the chemical structures of the compounds. (**e**) Relative cell numbers to day 0 of BON-1 or QGP-1 cells treated with vehicle or CPI203 over a 10-day period. Error bars represent S.E.M., *n*=3. Population doubling time of each treatment was listed in the table underneath the cell proliferation curve. (**f**) Cell cycle analysis at 48 h of BON-1 or QGP-1 cells treated with CPI203, (+)-JQ1, PFI-1 or (−)-JQ1 as indicated. (**g**) to (**j**) Knockdown of BET protein BRD4 prevented cell proliferation in BON-1 cells. (**g**) and (**h**) mRNA levels (**g**) or protein levels (**h**) of BET family genes in BON-1 cell line at 48 h after transfection with 25 nM SMARTpool siRNA oligos against NTC, *BRD2*, *BRD3* and *BRD4.* Error bars represent S.E.M., *n*=3. (**i**) Relative cell numbers to NTC of the siRNA transfected BON-1 cells of the above samples at 24 h, 48 h and 72 h. Error bars represent S.E.M., *n*=3. (**j**) Cell cycle profiles of the samples in (**i**). Error bars represent S.E.M., *n*=3

Two available human PanNET cell lines, BON-1 and QGP-1, and a bronchial NET cell line, NCI-H727 (H727), were incubated for 72 hours (h) with a range of concentrations of BETi CPI203. Of the three NET cell lines, the BON-1 cell line was the most sensitive to CPI203 (Figure 1a) with a half-maximal growth inhibitory concentration (GI_50_) of 45 nM, whereas QGP-1 showed a little more sensitivity to CPI203 than H727 since the inhibition began to plateau at around 156 nM. To confirm the role of BETi in NET cell growth, NET cell lines were treated with two other BET inhibitors (+)-JQ1 and PFI-1 that displayed strong potency and specificity toward the acetyl-binding cavity of BET protein bromodomains.^13,15^ In agreement with the CPI203 data, BON-1 cells were most sensitive to (+)-JQ1 and PFI-1 with GI_50_ values 120 nM and 1.5 *μ*M (Figures 1b and c). In addition, cells were also treated with (+)-JQ1's inactive isomer (−)-JQ1.^13,16^ Both BON-1 and QGP-1 cells showed no responses to (−)-JQ1 up to 20 *μ*M, and H727 cells showed no responses to (−)-JQ1 up to 10 *μ*M but 50% growth inhibition at 20 *μ*M (Figure 1d). To further analyze cell proliferation inhibition, BON-1 and QGP-1 cells were treated with 50 nM, 100 nM, 500 nM or 2.5 *μ*M CPI203, and cell numbers were evaluated over a 10-day period. CPI203 inhibited cell proliferation of both cell lines within three days (Figure 1e). The inhibition of cell proliferation by CPI203 is dose-dependent as indicated by the proliferation curves and the population doubling time.

Cell cycle profile analysis of BON-1 and QGP-1 cells at 48 h after treatments with CPI203, (+)-JQ1 and PFI-1 but not (−)-JQ1 revealed increased G1 cells (Figure 1f). The pattern of increased G1 cells by BETi in PanNET cell lines is similar to that of cell proliferation inhibition in Figures 1a–d, indicating that growth inhibition by BETi led to G1 cell cycle arrest. Correspondingly, the protein level of cyclin-dependent kinase (CDK) inhibitor p27^KIP1^ (Kinase Inhibitory Protein 1) was increased at 48 h upon CPI203 treatment in both BON-1 and QGP-1 cells (Supplementary Figure S1a). Annexin-V positive cells and poly ADP ribose polymerase (PARP) cleavage were not detected after 72 h treatment (data not shown), suggesting that CPI203 treatment may not trigger apoptosis in PanNET cells. In the aggregate, BETi mainly prevented cell proliferation with cytostatic but not cytotoxic effects.

To assess the role of BET proteins in PanNET cell proliferation, siRNA technology was used to knockdown the three BET proteins, BRD2, BRD3 and BRD4 in BON-1 cells. Non-targeting control (NTC) siRNA was used as a negative control. Knockdown of each gene and protein with siRNA was confirmed by qPCR and immunoblots (Figures 1g and h). Relative to NTC, knockdown of BRD2 and BRD3 showed no more than 20% growth inhibition of BON-1 cells while knockdown of BRD4 showed 50% growth inhibition at 72 h (Figure 1i). Consistently, cell cycle profiles demonstrated the same pattern (Figure 1j). This suggested that knockdown of BRD2 and BRD3 had only small effects on BON-1 cell growth, and that growth inhibition of PanNET cells might be mainly due to the inhibition of BRD4 protein bromodomain upon BETi treatment.

### BETi-induced downregulation of MYC contributed to growth inhibition

**Figure 2:**
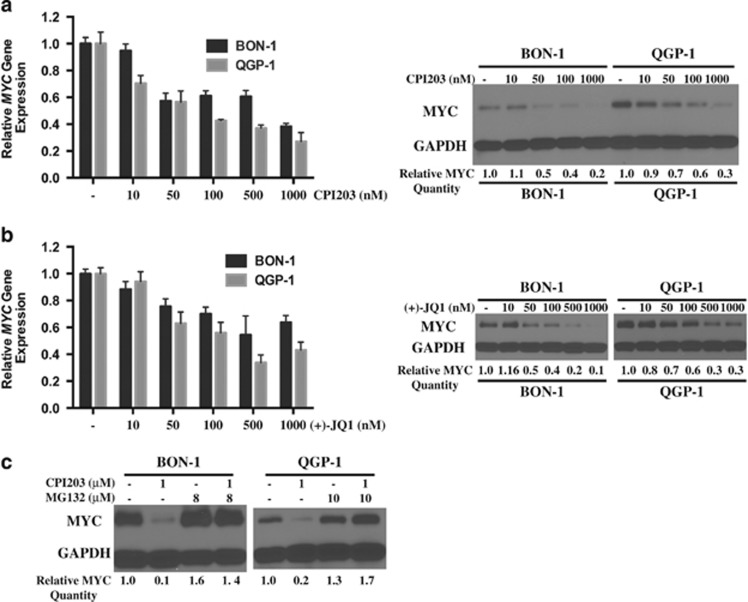

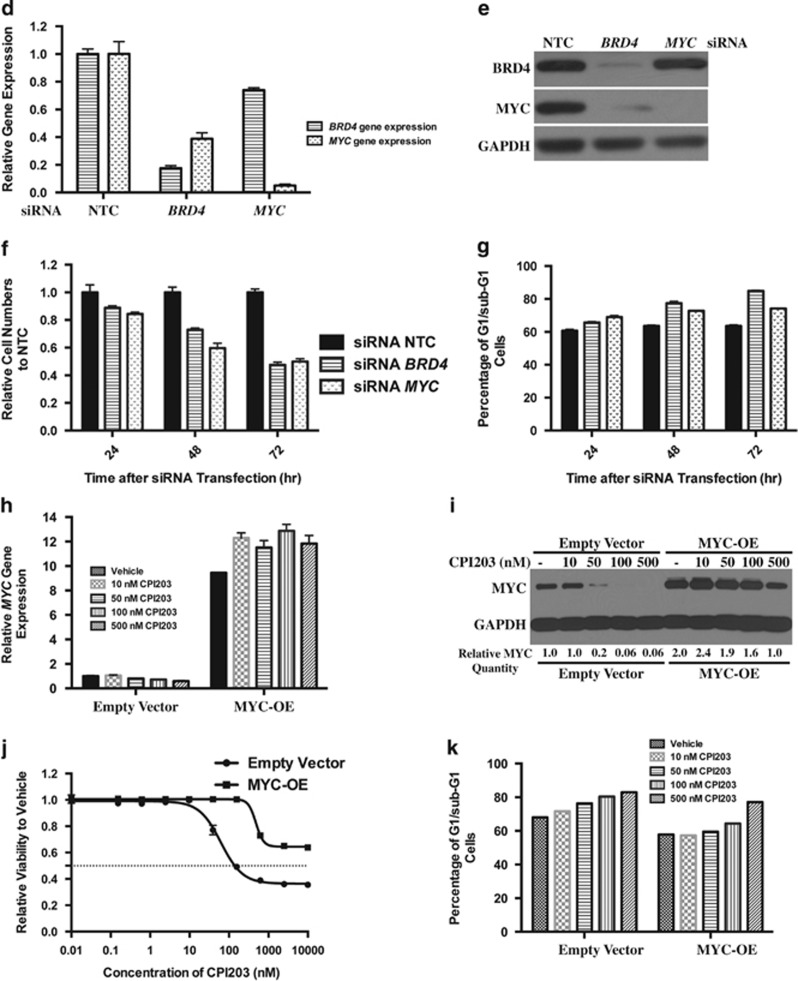
BETi-induced downregulation of MYC contributed to growth inhibition. (**a**) Quantitative real-time PCR analysis of *MYC* transcript and immunoblots of MYC protein level at 24 h in BON-1 and QGP-1 cells treated with CPI203 as indicated. Gene expression data were normalized to vehicle control. Error bars represent S.E.M., *n*=3. Relative MYC protein quantity was normalized to vehicle and shown at the bottom of the blots. (**b**) Quantitative real-time PCR analysis of *MYC* transcript and immunoblots of MYC protein level at 24 h in BON-1 and QGP-1 cells treated with (+)-JQ1 as indicated. (**c**) Proteasome inhibitor MG132 restored CPI203-reduced MYC protein level in PanNET cells. Relative quantification of MYC protein to vehicle control was shown on the bottom of the image. (**d**–**g**) siRNA knockdown of MYC prevented cell proliferation. (**d**) and (**e**) mRNA levels (**d**) or protein levels (**e**) of MYC and BRD4 treated with 25 nM SMARTpool siRNA oligos against NTC, *MYC* or *BRD4*. Error bars represent S.E.M., *n*=3. (**f**) Relative cell numbers to NTC of the siRNA transfected BON-1 cells in the above samples at 24 , 48 and 72 h. Error bars represent S.E.M., *n*=3. (**g**) cell cycle profiles of samples in (**f**). Error bars represent S.E.M., *n*=3. (**h**) to (**k**) exogenous expression of MYC rescued CPI203-induced growth inhibition. (**h**) and (**i**) quantitative real-time PCR analysis of *MYC* transcript (**h**) and immunoblots of MYC protein level (**i**) at 24 h in BON-1 cells with overexpression of MYC (MYC-OE) or an empty vector treated with DMSO or CPI203 as indicated. Error bars represent S.E.M., *n*=3. Relative MYC protein quantity was normalized to vehicle-treated BON-1 cells with empty vector and shown at the bottom of the blots. (**j**) Dose-response curves of BON-1 cells with MYC-overexpression or empty vector, treated for 72 h with DMSO or CPI203 in nine-point four-fold dilutions starting at 10 *μ*M. Error bars represent S.E.M., *n*=3. (**k**) Cell cycle analysis of BON-1 cells with MYC-overexpression or empty vector, treated for 48 h with DMSO or CPI203 as indicated

It has been reported that (+)-JQ1 reduces *MYC* in lymphoma and leukemia cell lines through BRD4 protein bromodomain inhibition, and deregulated expression of MYC exerts significant roles on cell cycle progression.^17^ Figure 2a showed that at 24 h upon CPI203 treatment, *MYC* mRNA was downregulated by about two- or three-fold in BON-1 or QGP-1 cells respectively, and MYC protein was reduced in a dose-dependent manner with a slightly stronger reduction of MYC protein in BON-1 cells than in QGP-1 cells. Similar pattern of MYC downregulation was also observed in (+)-JQ1-treated BON-1 and QGP-1 cells (Figure 2b). Since BRD4 has been implicated in stabilizing nuclear NF-*κ*B by preventing the ubiquitination of RELA,^18^ we interrogated whether CPI203 treatment regulated MYC stability. BON-1 and QGP-1 cells were treated with 1 *μ*M CPI203 for 24 h in the presence or absence of the proteasome inhibitor MG132 and we found that MG132 abolished CPI203-induced MYC protein downregulation in both cell lines (Figure 2c). This suggested that BETi might regulate MYC stability and underlined that BRD4 might regulate cellular proteins not only at the transcriptional but also at the post-transcriptional level.

We then examined whether reduced MYC expression would phenocopy the effects of BETi treatment in BON-1 cells. siRNA knockdown of MYC and BRD4 was confirmed by qPCR and immunoblots (Figures 2d and e). BRD4 knockdown caused reduction of *MYC* mRNA and protein, indicating that BRD4 regulates *MYC* gene expression in BON-1 cells. Knockdown of MYC inhibited cell growth and increased G1 cells, and the level was similar to that of BRD4 knockdown (Figures 2f and g), demonstrating that downregulation of MYC phenocopied the effects of BETi treatment on BON-1 cells. We further determined whether exogenous expression of *MYC* could rescue BON-1 cells from the CPI203-induced growth inhibition. BON-1 cells were stably transduced with a retroviral vector expressing *MYC* (MYC-OE) or an empty vector. The overexpression of MYC was confirmed by qPCR and immunoblots (vehicle-treated bands or bars in Figures 2h and i). These cells were treated with a range of doses of CPI203. As seen in Figures 2j and k, MYC overexpression rescued cells from CPI203-induced growth inhibition and cell cycle arrest.

### CPI203 treatment decreased MYC target gene expression

**Figure 3:**
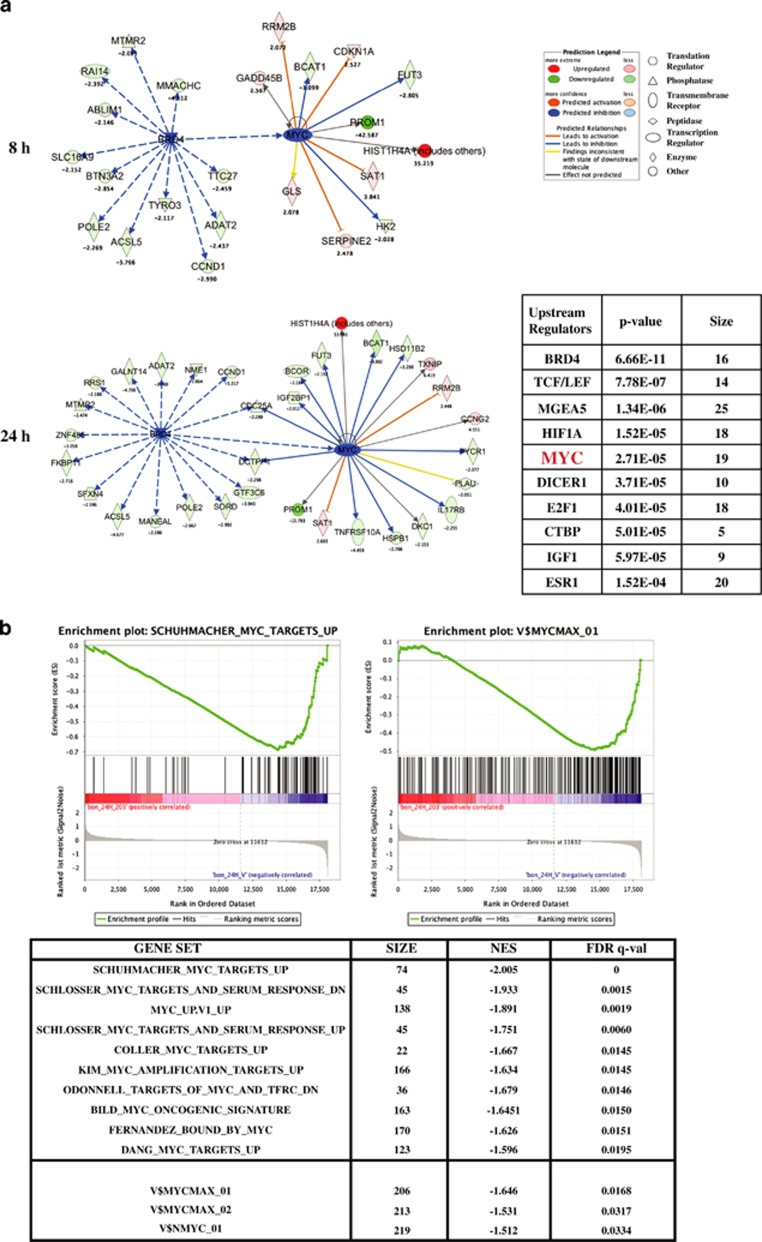
CPI203 treatment decreased MYC target gene expression. (**a**) Ingenuity Pathway Analysis (Ingenuity Systems) identified BRD4 as the most significant upstream regulator and MYC as the fifth significant upstream regulator of the differentially expressed genes at 24 h, and BRD4 regulates MYC. Left panel showed the genes regulated by BRD4 and MYC at 8 h and 24 h. The table on the bottom right panel listed the top ten significant upstream regulators of the differentially expressed genes at 24 h. (**b**) Genome-wide transcriptome analysis of BON-1 cells treated with 1 *μ*M CPI203 identified significant 15 MYC-target gene sets and MYC binding site gene sets. NES indicates normalized enrichment score and q means false discovery rate. Top panel are the representative plots of gene set signatures. Lower panel is the list of top ten MYC target gene set signatures

Since MYC is a TF that controls gene expression programming mediating cell growth, proliferation and survival, we investigated whether transcription of MYC target genes were affected upon CPI203 treatment. A microarray analysis was performed on BON-1 cells collected at 8 and 24 h upon exposure to 1 *μ*M CPI203. The pattern of gene expression changes at 8 h is mostly similar to that at 24 h. As expected, *MYC* mRNA was downregulated by a 1.5-fold change with *P*-value 0.005. The list of differentially expressed genes (DEgenes) at 8 and 24 h (Supplementary Table S1 and S2) upon CPI203 treatment was examined for upstream transcriptional regulators using Ingenuity Pathway Analysis (IPA) (Ingenuity Systems, Redwood City, CA, USA; www.ingenuity.com). BRD4 was the most significant upstream regulator at 8 h and 24 h and MYC was the fifth significant regulator of those differentially expressed genes at 24 h upon CPI203 treatment (Figure 3a). Alternatively, gene set enrichment analysis (GSEA)^19,20^ with multiple corrections (FDR <0.05) using the gene expression data was performed to identify curated gene set signatures and TF target gene set signatures. Curated gene set analysis on all MYC gene sets (51 MYC gene sets in MSigDB_v3.0)^20,21^ revealed that 15 MYC gene sets were downregulated in CPI203-treated cells (Figure 3b) and downregulation of these MYC gene sets was stronger at 24 h than at 8 h. GSEA using TF motif signature from MSigDB was also carried out. Gene set signature described by MYC binding sites (V$MYCMAX_01, V$MYCMAX_02 and V$NMYC_01) was significantly enriched for CPI203-affected genes (Figure 3b). Thus, downregulation of MYC by CPI203 caused deregulation of MYC-target genes, as expected. In sum, MYC plays a critical role in the regulation of cell proliferation of PanNET cells.

### BETi enhanced the growth inhibition of rapamycin and reduced the rapamycin-induced activation of AKT in PanNET Cells

**Figure 4:**
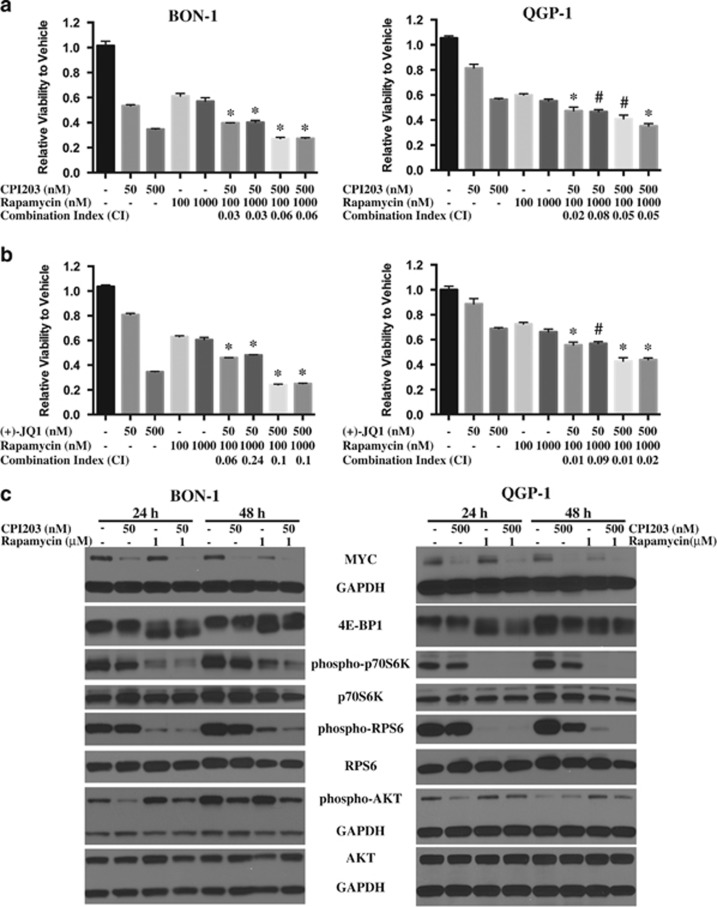

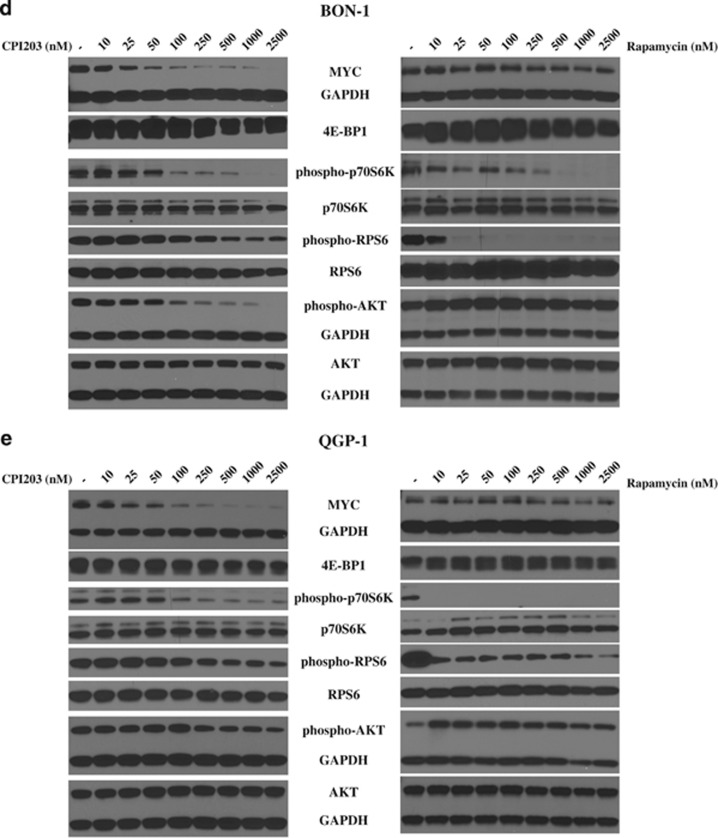
BETi enhanced the growth inhibition of rapamycin and reduced the rapamycin-induced activation of AKT in PanNET Cells. (**a**) Synergistic growth inhibition of CPI203 and rapamycin on BON-1 or QGP-1 cells. BON-1 or QGP-1 cells were treated with CPI203, rapamycin or in combination as indicated. Cell viability was determined at 72 h. Error bars represent S.E.M., *n*=3. Combination index (CI) was calculated using CompuSyn software. *P<0.01 *versus* single treatment; ^#^*P*<0.01 *versus* one single treatment but *P*=0.01 *versus* the other single treatment. (**b**) Synergistic growth inhibition of (+)-JQ1 and rapamycin on BON-1 or QGP-1 cells, which was performed in the same way as that in (**a**). (**c**) Immunoblots of MYC, mTOR downstream targets and AKT at 24 and 48 h of BON-1 and QGP-1 cells treated with 50 nM CPI203 (BON-1) or 500 nM (QGP-1), 1 *μ*M rapamycin or in combinations. (**d**) and (**e**) Immunoblots of MYC, mTOR downstream targets and AKT at 48 h of BON-1 cells (**d**) or of QGP-1 cells (**e**) treated with a range of doses of CPI203 or rapamycin as indicated

Rapamycin (sirolimus) and analogues are specific inhibitors of mTORC1 and demonstrated a broad-spectrum antitumor activity both *in vitro* and *in vivo* with an acceptable safety profile.^22^ It has also been reported that rapamycin treatment showed limited clinical efficacy, which may be due to the feedback activation of AKT triggered by mTORC1 inhibition.^23,24^ Combination of rapalogs with other anticancer drugs might improve efficacy. Since everolimus is an FDA-approved treatment option for NETs, we sought to explore the growth inhibitory effects of BETi in combination with rapamycin as well as its underlying mechanisms in PanNETs. BON-1 or QGP-1 cells were treated with 50 or 500 nM CPI203 alone, 100 nM or 1 *μ*M rapamycin alone, or in combinations. For both cell lines, all the combination treatments were more effective at blocking cell growth than either agent alone (*P*<0.01 or *P*=0.01) (Figure 4a). Both cell lines were also treated with a range of doses of CPI203 and rapamycin alone at the same time (data not shown). According to the Chou–Talalay method,^25^ the combination index (CI) was calculated using CompuSyn software^26^ (ComboSyn Inc.; Paramus, NJ, USA) for each combination and CIs were less than 1 for all the combination treatments in both cell lines, indicating synergistic effects of CPI203 and rapamycin in both cell lines. The synergistic effects of (+)-JQ1 and rapamycin were observed in both cell lines as well (Figure 4b). These indicated that co-treatment of BETi and rapamycin resulted in synergistic effects on cell proliferation in PanNET cells. Consistent with the synergistic growth inhibition, co-treatment of CPI203 and rapamycin led to stronger G1 cell cycle arrest at 48 h (Supplementary Figure S1b), but not apoptosis. Annexin-V positive cells and PARP cleavage were not detected at 72 h upon co-treatment of CPI203 and rapamycin (Supplementary Figure S1c).

We then sought to explore the mechanism of synergy between BETi and mTOR inhibitor. BON-1 and QGP-1 cells were treated with CPI203 (50 nM on BON-1 or 500 nM on QGP-1), 1 *μ*M rapamycin or in combination and the protein lysates were collected at 24 and 48 h after treatments. Both cell lines were treated with a range of doses of CPI203 or rapamycin alone at the same time and the protein lysates were collected at 48 h after treatments. It has been reported that rapamycin limits MYC protein expression by inhibiting *MYC* mRNA translation.^27^ As reported, MYC was downregulated upon more than 250 nM rapamycin treatment in both BON-1 and QGP-1 cells (Figures 4d and e). The combination treatment enhanced CPI203-induced MYC downregulation (Figure 4c), suggesting that the synergistic effect of BETi and rapamycin is in part related to the synergistic decrease of MYC protein.

In addition, the levels of the key proteins in the mTOR downstream pathways and their post-translational modifications were studied as possible important players in the synergistic effects described above. We interrogated the phosphorylation status of the downstream targets of mTORC1, such as p70 S6 kinase (p70S6K), ribosomal protein S6 (RPS6) and eukaryotic initiation factor (eIF4E) binding protein 4E-BP1 in both PanNET cell lines. As seen in Figures 4c, d and e, rapamycin treatment inhibited phosphorylation of p70S6K, RPS6 and 4E-BP1 as reported. CPI203 treatment led to a decreased expression of total 4E-BP1 and inhibition of the phosphorylation of p70S6K and RPS6. These effects were dose-dependent and CPI203 did not reduce total p70S6K and RPS6 proteins. The inhibitory activities were further enhanced in the combination treatment in both cell lines (Figure 4c) and these inhibitory effects were also observed in two other combinations of CPI203 and rapamycin treatment (data not shown).

The limitation of rapamycin treatment in cancer therapeutics is linked to its upregulation of AKT through the negative feedback loop induced by mTORC1 inhibition. The phosphorylation status of AKT was investigated as well. Rapamycin induced phosphorylation of AKT in BON-1 and QGP-1 cells as expected, whereas CPI203 inhibited AKT phosphorylation and this inhibition was stronger in BON-1 cells than in QGP-1 cells (Figures 4c, d and e). Interestingly, CPI203 treatment reduced AKT phosphorylation induced by rapamycin (Figure 4c), implying that the BETi inhibitory effect is sufficient to block the feedback loop induced by mTORC1 inhibition.

### Combination treatment of CPI203 and rapamycin suppressed tumor growth *in vivo*

**Figure 5:**
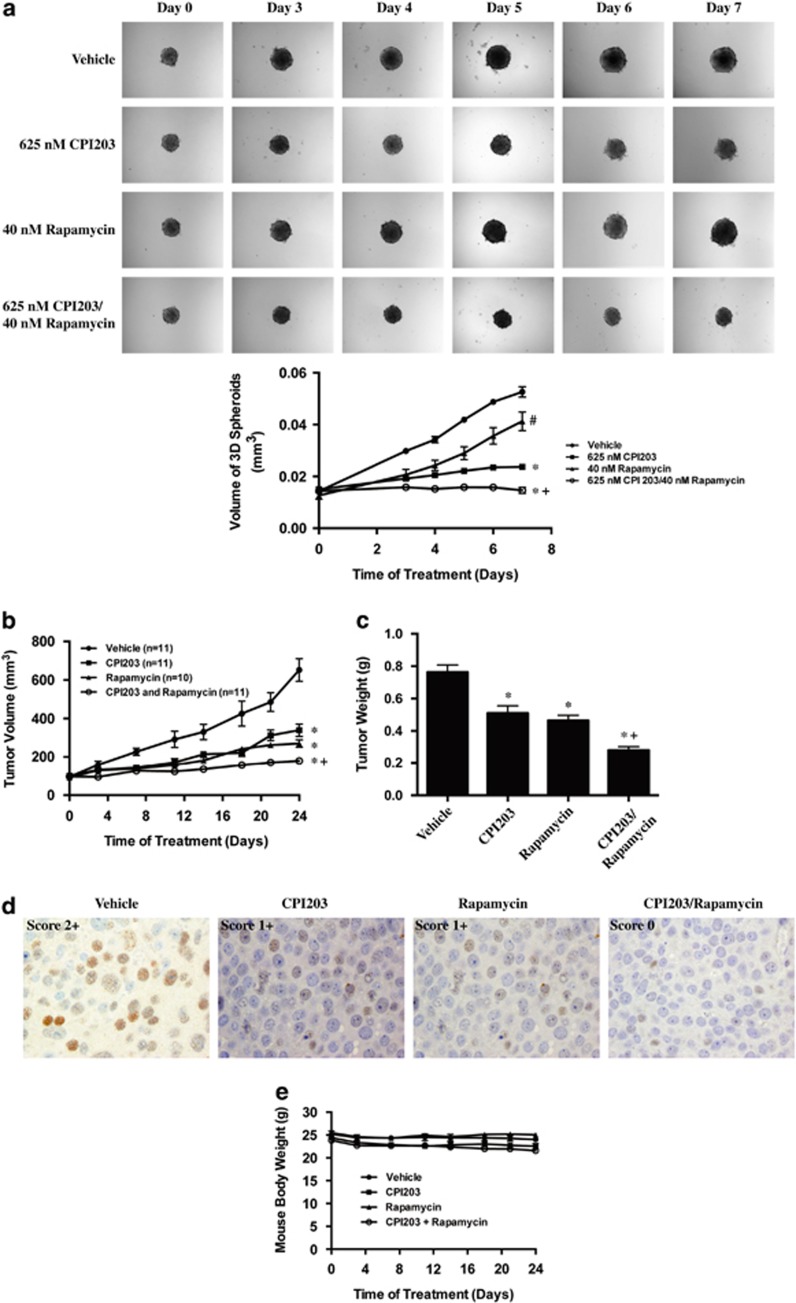
Combination treatment of CPI203 and rapamycin suppressed tumor growth *in vivo*. (**a**) Significant growth inhibition of CPI203 and rapamycin treatment on BON-1 3D tumor spheroids. BON-1 3D tumor spheroids were grown on 96-well plates coated with agarose and treated with 625 nM CPI203, 40 nM rapamycin or in combination. Size of the 3D spheroids was measured and calculated using SpheroidSizer program. Top panel showed the representative images of 3D tumor spheroids. Lower panel showed the growth curve of 3D spheroids after treatments. Error bars represent S.E.M., *n*=8. (**b**) Significant growth inhibition of CPI203 and rapamycin treatment on BON-1 xenograft tumors. Subcutaneous BON-1 xenograft tumors were treated with CPI203 (5 mg/kg, *n*=11), rapamycin (15 mg/kg, *n*=10) or in combination (*n*=11) when tumor size reached around 100 mm^3^. (**c**) Tumor weight values were significantly smaller in the mice with the combination treatments. Tumors were excised at the end of the treatment period and weighed. (**d**) MYC protein expression in the above treated BON-1 xenograft tumors. Representative images of MYC immunohistochemistry in the treated tumors as indicated. All panels are in the same magnification. (**e**) Body weight of mice during treatments showed that the treatments were not toxic. Mice were weighed twice per week for 3.5 weeks to monitor the toxicity. ^#^*P*=0.01 *versus* vehicle control; *P<0.01 *versus* vehicle control; ^+^*P*<0.01 *versus* both single treatment

To investigate whether rapamycin and CPI203 inhibited cell proliferation *in vivo*, we first tested the compounds in a three-dimensional (3D) BON-1 tumor spheroids assay. It has been reported that 3D tumor spheroids better mimick *in vivo* tumor architecture and better predict *in vivo* efficacy.^28^ BON-1 3D tumor spheroids were grown as described,^29^ treated with 625 nM CPI203, 40 nM rapamycin or in combination, and the 3D tumor spheroids were imaged at day 3, 4, 5, 6 and 7 following treatment. The size of the 3D tumor spheroids were measured using SpheroidSizer program as reported.^30^ Combination treatment of rapamycin and CPI203 caused a statistically significant inhibition of cell growth on 3D BON-1 tumor spheroids (Figure 5a).

Next we investigated the antitumor activity of CPI203, rapamycin and the combination *in vivo*. Human BON-1 xenografts were established subcutaneously in nude mice, which were treated for 24 days with CPI203 (5 mg/kg), rapamycin (15 mg/kg) or the combination. CPI203 or rapamycin treatment alone showed significant inhibition of tumor growth compared to control (Figure 5b). Notably, combination treatments with CPI203 and rapamycin significantly inhibited tumor growth compared to control or individual treatments with *P*-values less than 0.01. The tumor weights at time of sacrifice confirmed the tumor volume results (Figure 5c). Immunohistochemical staining indicated that MYC protein expression was decreased in both CPI203 and rapamycin single agent-treated tumors and was further decreased in the tumors with combination treatment (Figure 5d). This suggested that the enhanced tumor growth inhibition might be in part due to an enhanced inhibition of MYC levels. Of note, 5 mg/kg CPI203 treatment for 14 days led to slight body weight loss, the dose was then reduced to 3.5 mg/kg for single and combination treatments for the remaining 10 days and there was no significant reduction in body weight (Figure 5e). Taken together, these data indicated that the dose and treatment schedule tested in this experiment were well tolerated by the mice and demonstrated that the therapeutic potential of targeting endogenous MYC in PanNET tumors through the use of BETi and rapamycin.

We further investigated whether the antitumor mechanism could be due to apoptosis *in vivo*. Immunohistochemical staining of cleaved caspase-3 indicated that treatment of CPI203, rapamycin or in combination did not increase the degree of apoptosis in BON-1 xenograft tumors and immunoblotting did not show increased PARP cleavage in the treated tumors either (data not shown). In addition, tumor regression was not observed in the treated tumors. All these suggested that apoptosis might not be the mechanism of BON-1 xenograft tumor growth inhibition by CPI203 or/and rapamycin.

## Discussion

**Figure 6:**
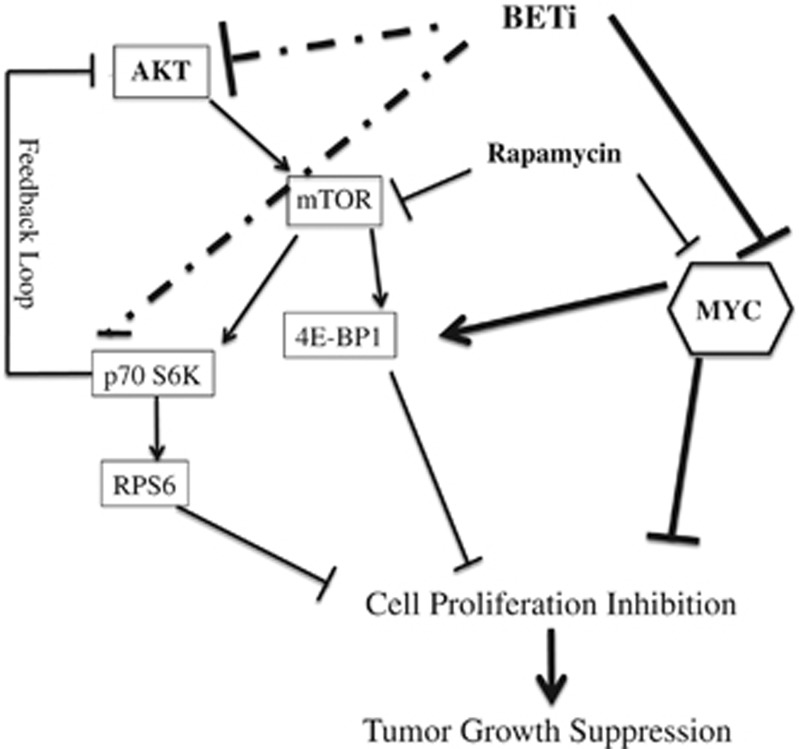
Regulation of cell growth by MYC and mTOR pathway in the PanNETs. Schematic representations of mTOR pathway and MYC in cell growth and proliferation in PanNETs were shown. In this study, MYC was mainly inhibited with BETi CPI203 and mTOR pathway was inhibited by rapamycin. Thick solid lines showed interactions from this study in PanNETs and other studies; dotted lines showed interactions from this study. Thin lines showed the known interactions. p70S6K: ribosomal protein S6 kinase; 4E-BP1: eIF4E binding protein 1; RPS6: ribosomal protein S6

*MYC* gene amplification or deregulation is estimated in up to 70% of human cancers and has been linked to the tumorigenicity of many cancers.^31^ The emerging role of BET proteins as the inhibitors of MYC suggests that the BET protein BETi may become a therapeutic option for these cancers. Although MYC is not frequently deregulated in PanNETs, several observations suggested that MYC activation appeared to be an early event in human islet cell tumor progression.^32,33^ Studies in transgenic mouse models with conditional *Myc* expression showed that *Myc* gene expression is not only required for the initiation of PanNETs but also important in maintaining the tumorigenicity of PanNETs.^7,34^ In this study, we extended these observations and confirmed that downregulation of MYC protein in PanNET cells upon BETi treatment inhibited cell proliferation and had antitumor effects *in vivo*. Moreover, co-treatment of CPI203 and rapamycin enhanced the antiproliferative effects of mTOR inhibition *via* a stronger MYC protein downregulation and inactivation of mTOR downstream targets (Figure 6). Additionally, we found that BETi inhibited the activation of phospho-AKT and could overcome the rapamycin-induced AKT activation. Finally, we demonstrated that combination treatment of CPI203 and rapamycin repressed BON-1 xenograft tumor growth *in vivo*, suggesting that combination treatment of BETi and rapalog may provide a viable therapeutic strategy for treatment of PanNETs and other tumors.

BET inhibitors have been widely studied to downregulate MYC in myc-overexpressed cancers and to have anticancer effects.^16,35^ In this paper, we demonstrated that BETi exhibited antitumor effects by downregulating endogenous MYC in PanNET cells *in vitro* and *in vivo*. siRNA knockdown of BRD4 indicated that BRD4 regulates *MYC* gene expression in PanNET cells. CPI203 treatment led to about two-fold reduction of *MYC* mRNA and about five-fold reduction of MYC protein in BON-1 cells. Proteasome inhibitor MG132 stabilized MYC protein level in the presence of CPI203, suggesting that CPI203 or BRD4 might regulate the stability of MYC protein as well. This phenomena was described in B-precursor acute lymphoblastic leukemia cells where (+)-JQ1 treatment decreases MYC stability.^36^ Further experiments need to be designed to determine how BETi regulates MYC at the post-transcriptional level. Although MYC level is critical for cell proliferation in PanNET cells, factors other than MYC may also contribute to the cell proliferation inhibition. CPI203 treatment of MYC-knockdown BON-1 cells showed slightly reduced cell proliferation and slightly increased G1/sub-G1 cells (Supplementary Figure S2). Besides, global gene expression analysis, qPCR assays and western blots analyses identified CPI203-induced downregulation of *CCND1*, *HAS2, hTERT*, *BCL-XL*, *AURKA/B*, and other genes/proteins involved in cell proliferation, cell cycle and tumorigenesis (data not shown). Further analyses on how these genes/proteins contribute to NET cell proliferations will help understand the tumorigenesis of NET.

It has been reported that 14% of PanNETs bear mutations in genes involved in the PI3K/mTOR pathway,^37^ stressing the importance of PI3K/mTOR pathway in NET tumorigenicity. Indeed rapalogs have been widely used as a treatment option for NET patients. Here we demonstrated that combination treatment of CPI203 with rapamycin repressed PanNET cell growth synergistically *in vitro* and *in vivo*. The molecular mechanism could be due to enhanced MYC protein downregulation and/or enhanced inactivation of mTOR downstream targets and/or inactivation of rapamycin-induced AKT (Figure 6). We showed that CPI203 decreased expression of 4E-BP1 in PanNET cells potentially *via* MYC downregulation, as reported.^38,39^ Further, we discovered that CPI203 reduced phophorylation of p70S6K, RPS6 and AKT in a CPI203 dose-dependent manner, which may contribute to cell growth inhibition as well. Global gene expression profiling data showed that *p70S6K*, *RPS6*, *AKT1*, *2* and *3* mRNA level did not change at 24 h upon CPI203 treatment and immunoblots showed that total p70S6K, RPS6 and AKT protein levels did not change either. We did find that CPI203 inactivated the phosphorylation of other kinases of MAPK pathways (data not shown). Thus it would be possible that lowered phosphorylation of AKT upon CPI203 treatment could be owing to the regulation of growth factor receptor genes by BRD4 or MYC. In accord with our data, it has been reported that MYC amplification abrogated sensitivity to PI3K- or mTOR- targeted therapy due to activation of AKT.^40,41^ The exact mechanism of how BETi interferes with the activation of AKT and p70S6K is worth pursuing. PI3K/AKT/mTOR has been frequently mutated in other cancers and rapamycin has been often used clinically.^42,43^ If targeting endogenous MYC and mTOR pathway can counteract the limitation of rapamycin, the combination treatment of rapalogs with BETi will be beneficial not only in NETs but also in other cancers.

## Materials and Methods

### Cell lines and culture conditions

Human pancreatic neuroendocrine tumor cell lines BON-1 (provided by Kjell Oberg, Uppsala University, Sweden), QGP-1 (Japanese Health Sciences Foundation, Osaka, Japan) and human lung neuroendocrine tumor cell line NCI-H727 (ATCC, Manassas, VA, USA) were used in this study. BON-1 and NCI-H727 cells were maintained in DMEM/F-12 (Dulbecco's Modified Eagle Medium/Nutrient Mixture F-12) medium (Life Technologies, Grand Island, NY, USA) and QGP-1 cells were maintained in RPMI-1640 medium (Life Technologies), supplemented with 10% certified FBS (Life Technologies) and 0.1 mg /ml primocin solution (Invivogen, San Diego, California, USA). All cells were kept in a 37 °C humidified incubator with 95% air and 5% CO_2_.

### Compounds

CPI203 was supplied by Constellation Pharmaceuticals Inc. (Cambridge, MA, USA), (+)-JQ1, (−)-JQ1 and PFI-1 were purchased from Cayman Chemical Inc. (Ann Arbor, MI, USA), and rapamycin was purchased from LC Laboratories Inc. (Woburn, MA, USA).

### Cell proliferation assays

Cells were seeded in 96-well culture dishes at a density of 5000 cells per well for BON-1 and QGP-1, 4000 cells per well for NCI-H727 at the day before treatment and treated with the compounds as indicated. Drugs were dissolved in DMSO and nine-point four-fold dilutions starting at 20 or 10 *μ*M were made in the complete growth media and subsequently added in a triplicate to the wells of 96-well plates. 72 h after drug treatments, CellTiter 96 Aqueous One Solution Cell Proliferation Assay (Promega, San Luis, CA, USA) was used to detect the viable cells per manufacturer's instructions. Each experiment was performed three times independently. GI_50_ values were calculated with GraphPad Prism (GraphPad Software, La Jolla, CA, USA). Combination Index (CI) for drug interaction (e.g., synergism) was calculated using the CompuSyn software (ComboSyn, Inc).^26^ According to Chou–Talalay method, if CI is less than 1, the two drugs show synergism; if CI is greater than 1, the two drugs show antagonism.^25^

For cell number counting assay, 120 000 BON-1 cells or 150 000 QGP-1 cells were plated onto 12-well plates at the day before treatment. Cell numbers and viability were assessed on day 1, 3, 5, 7 and 10 (for BON-1 cells) or on day 1, 3, 6, 8, and 10 (for QGP-1 cells) after the treatments using the Millipore Guava Easycyte-5HT (Millipore, Billerica, MA, USA) or the Vi-cell analyzer (Beckman Coulter, Brea, CA, USA) per manufacturers' manuals. Experiments were performed in triplicate and each experiment was repeated three times independently.

### Determination of population doubling time (PDT)

Cell proliferation was determined by characterizing the log phase growth with population doubling time (PDT) which was calculated by using the equation: T * ln (2)/ln (Xe/Xb), where T is the incubation time; Xb is the cell number at the 24 h after seeding; Xe is the cell number at the 72 h after seeding. Cell numbers were counted by Millipore Guava EasyCyte 5HT flow cytometer (Millipore) or Vi-cell analyzer (Beckman Coulter).

### Cell cycle analysis

Cells were seeded at 350 000 cells per well onto 6-well plates, treated the next day with the compounds as indicated. At each time point, media was then collected from each individual well and cells were trypsinized for 3 min in the tissue culture incubator. Upon centrifugation at 1000 r.p.m. for 5 min, cell pellet was fixed in ice cold 70% ethanol and stored at 4 °C for at least 24 h. On the day of analysis, the fixed cells were treated with RNase A (Invitrogen, Grand Island, NY, USA), stained with Propidium Iodide (PI) (Invitrogen), and analyzed on a Beckman Coulter FC500 Analyzer (Beckman Coulter) using CXP analysis software (BD Biosciences, San Jose, CA, USA). All experiments were performed at least twice independently.

### Protein extractions and western blotting

Cells were harvested for proteins on ice at different time points using RIPA lysis buffer (Thermo Scientific, Rockford, IL, USA) with the addition of PhosStop and Protease inhibitor cocktail tablets (Roche, Indianapolis, IN, USA), cleared by centrifugation for 20 min at 13 000 r.p.m., and protein concentrations were quantified with the BCA TM protein assay kit (Thermo Scientific). Protein samples were boiled in SDS loading buffer, resolved on 12% Bio-Rad gel, and transferred to PVDF membranes (Millipore). Membranes were incubated overnight at 4 ^o^C with the primary antibodies. In the following day, membranes were incubated with goat anti-rabbit or anti-mouse secondary antibody (1 : 4000, Santa Cruz Biotechnology Inc., Dallas, Texas, USA). SuperSignal West Pico Chemiluminescent Substrate (Thermo Fisher, Waltham, MA, USA) was used for the detection of the primary antibody bound to the antigen on the membrane. Rabbit polyclonal anti-GAPDH antibody was purchased from Santa Cruz Biotechnology, Inc.. The following antibodies were purchased from Cell Signaling Technology Inc. (Danvers, MA, USA): MYC, AKT, p-AKT (S473), p-RPS6 (S235/236), RPS6, p-4EBP1 (S65), 4EBP1, p-p70S6K, p70S6K. Antibodies against BRD 2 and 3 were purchased from Bethyl Laboratories, Inc. (Montgomery, TX, USA). Antibody for BRD4 was purchased from Abcam (Cambridge, MA, USA). GAPDH protein was used as a loading control for immunoblots in all the experiments. All the experiments were repeated at least twice independently.

### RNA extractions and quantitative RT-PCR

Total RNA was isolated using the RNeasy Mini kit (Qiagen, Valencia, CA, USA) per manufacturer's instruction. cDNA was synthesized from 1 *μ*g of Total RNA using TaqMan Reverse Transcription Reagents (Life Technologies) per manufacturer's instruction. Real-time PCR was performed on an ABI 7500 instrument using TAQMAN Assay. *β*2 microglobulin (B2M) gene expression was always performed at the same time as an internal control. All experiments were performed in triplicates and each experiment was done at least twice independently.

### Retroviral transduction and stable cell line establishment

A bacterial construct encoding *MYC* cDNA in the pBabe-Zeo retroviral Vector (pBabe-*MYC*) was obtained from Addgene (Cambridge, MA, USA). Platinum A cells were cultured per manufacturer's protocol (Cell Biolabs Inc., San Diego, CA, USA). Platinum A cells were seeded into culture dishes at the day before transfection in DMEM/F12 medium supplemented with 10% FBS and 1% penicillin/streptomycin. Platinum A cells were transfected with pBabe-*MYC* or a control empty vector (pBabe-Zeo). Forty-eight hours after transfection, retroviral supernatants were collected and used to infect BON-1 cells seeded 24 h earlier. 24 h after infection, the viral medium was replaced with regular growth medium for BON-1 cells. Stable cell line was selected with 750 *μ*g/ml zeocin.

### siRNA transfection

120 000 BON-1 cells were seeded on 12-well plates at the day before transfection and were transfected with 25 nM ON-TARGET plus SMARTpool siRNA (Thermo Scientific) targeting each gene or a NTC using DharmaFECT 1 transfection reagent (Thermo Scientific) per well, according to the manufacturer's instructions. All experiments were performed in triplicates and each experiment was done three times independently.

### Gene expression profiling

BON-1 cells were treated with vehicle or 1 *μ*M CPI203 for 8 h or 24 h. Total RNA was isolated using the QIAGEN total RNeasy Mini Kit (Qiagen) and samples of 0.4 *μ*g RNA were subjected to Affymetrix gene chip U133 plus 2.0 analyses according to the manufacturer's protocol. The raw Affymetrix.CEL files were imported into GeneSpring GX11 software (Silicon Genetics, Redwood City, CA, USA) and did quality control. Normalized expression values were calculated by the GC Robust Multi-array Average (GCRMA) algorithm and subjected to mean transformation to collapsed all probes to respective genes in R. Statistical Analysis using student's *t*-test has been done to find differentially expressed genes with 2 Fold change and *P*-value 0.01 cut-off compared with the vehicle expression values. IPA (Ingenuity Systems) was performed using the differentially expressed genes according to the instructions from IPA. GSEA has been done on the expression values of all samples using GSEA software (Broad Institute, Cambridge, MA, USA) to find the statistically enriched gene sets between two biological states (phenotype). In GSEA analysis, we have used only MYC related gene sets as background to look for significant MYC related gene signatures. We used all default parameter to run GSEA for samples less than seven.

### NET cancer xenografts and treatments

The *in vivo* efficacy of CPI203, rapamycin or their combination was evaluated in BON-1 human NET tumor xenograft models, in accordance with the Institutional Animal Care and Use Committee (IACUC) guidelines of Rutgers University. Five- to six-week old female athymic (nu/nu) mice were ordered from NCI Mouse Repository (Frederick, MD, USA) and housed under pathogen-free conditions in microisolator cage with laboratory chow and water *ad libitum*. Human BON-1 cells were implanted subcutaneously into the flank region of female nude mice at 5 × 10^6^ cells per mouse in serum-free medium. When tumors reached a size of ~100 mm^3^, the tumor-bearing mice were randomized into four groups with a cohort size of 10–11 per group according to tumor volumes and body weights for the following treatments: Vehicle control, CPI203 (5 mg/kg; BID, I.P.), rapamycin (15  mg/kg, QWK, I.P.) and their combination. CPI203 was given 5 mg/kg daily by IP for 2 weeks and 3.5 mg/kg for the remaining 10 days. All treatments were given for 24 days. Tumor volumes were measured using digital caliper twice per week, calculated with the formula V=0.5 * (length x width^2^).

### Immunohistochemical assay

Paraffin-embedded tumor tissue blocks were used for immunohistochemical staining. MYC rabbit monoclonal antibody was obtained from Epitomics (Burlingame, CA, USA) and used at 1:200 dilution. Immunohistochemistry was performed on BenchMark XT automated equipment (Ventana Medical System Inc., Tucson, AZ, USA). Sections were pretreated with CC1 solution provided by Ventana followed by primary antibody incubation for 60 min, secondary antibody (1 : 200) incubation for 60 min, and chromogen development with DAB MAP Kit (Ventana, Tucson, AZ, USA). Positive control tissue was stained in parallel with all the study samples. MYC immunoreactivity was evaluated by the combination of staining intensity and the percentage of tumor cells with positive nuclear staining. The results are expression as in 4 tiers: score 3+ indicating moderate to strong staining intensity in >75% tumor cells, score 2+ indicating moderately to strong intensity in 50–75% tumor cells, score 1+ indicating moderate to weak intensity in 25–50% tumor cells, and score 0 indicating mostly weak staining intensity with <25% tumor cells.

### Statistical analysis

Graphs were produced using GraphPad Prism version 6.0b software. The statistical significance of differences between two groups or among multiple groups was analyzed with unpaired Student's *t* test (for equal variances) or with Welch's corrected *t* test (unequal variances) or one-way analysis of variance (ANOVA) by use of R software package. *P*<0.05 was considered to be statistically significant.

## Abbreviations

BET: Bromodomain and extra-terminal
BETi: BET protein bromodomain inhibitor
NETs: neuroendocrine tumors
PanNETs: pancreatic neuroendocrine tumors
rapalogs: rapamycin analogs
mTOR: mammalian target of rapamycin
3D: three-dimensional
MYC: c-MYC

## Notes

Emmanuel Normant and Peter Sandy are employees of Constellation Pharmaceuticals.
