# Editorial Expression of Concern: The bromodomain and extra-terminal inhibitor CPI203 enhances the antiproliferative effects of rapamycin on human neuroendocrine tumors

**DOI:** 10.1038/s41419-026-09313-9

**Published:** 2026-09-25

**Authors:** C. Wong, S. V. Laddha, L. Tang, E. Vosburgh, A. J. Levine, E. Normant, P. Sandy, C. R. Harris, C. S. Chan, E. Y. Xu

**Affiliations:** 1 Raymond and Beverly Sackler Foundation Laboratory, 195 Little Albany Street, New Brunswick, NJ 08901 USA; 2 Rutgers Cancer Institute of New Jersey, Robert Wood Johnson Medical School, Rutgers, The State University of New Jersey, 195 Little Albany Street, New Brunswick, NJ 08901 USA; 3 Department of Medicine, Robert Wood Johnson Medical School, Rutgers, The State University of New Jersey, 195 Little Albany Street, New Brunswick, NJ 08901 USA; 4 Center for Systems Biology, RobertWood Johnson Medical School, Rutgers, The State University of New Jersey, 195 Little Albany Street, New Brunswick, NJ 08901 USA; 5 Department of Pathology, Memorial Sloan-Kettering Cancer Center, New York, NY 10065 USA; 6 Department of Pediatrics, RobertWood Johnson Medical School, Rutgers, The State University of New Jersey, 195 Little Albany Street, New Brunswick, NJ 08901 USA; 7 School of Natural Sciences, Institute for Advanced Study, 1 Einstein Drive, Princeton, NJ 08540 USA; 8 Constellation Pharmaceuticals, Cambridge, MA 02142 USA

Editorial Expression of Concern to: *Cell Death & Disease* https://doi.org/10.1038/cddis.2014.396, published online 09 October 2014

The Editor-in-Chief is issuing an editorial expression of concern to alert readers that after the publication of this article, two image integrity concerns were raised. Specifically:

- In figure 5a, the panels for days 6 and 7 appear to overlap for the Vehicle
- In figure 5a, the panels for days 6 and 7 appear to overlap for 625 nM CPI203
- In figure 5d, CPI203 and Rapamycin appear to overlap with different colouration

The authors provided an explanation and original images but the time course experiment could not be verified for correction. Readers are urged to take caution when interpreting the content and conclusions of this article.

C. S. Chan agrees with this Editorial Express of Concern. A. J. Levine disagrees with this Editorial Express of Concern. C. Wong, S. V. Laddha, L. Tang, E Vosburgh, A. J. Levine, E. Normant, P. Sandy, C. R. Harris & E. Y. Xu did not respond to correspondence from the Publisher about this Editorial Express of Concern.

