# Supplementary material for: The bromodomain and extra-terminal inhibitor CPI203 enhances the antiproliferative effects of rapamycin on human neuroendocrine tumors
Source: Cell Death Dis. 2014 Oct 9;5(10):e1450–. doi: 10.1038/cddis.2014.396 (PMC4237236; doi:10.1038/cddis.2014.396)
Supplement: Supplementary Figure S1 [file cddis2014396x1.pdf]

Supplementary Figure legends

Supplementary Figure S1:

S1a. p27^{KIP1} protein expression was increased at 48 h upon CPI203 treatment: BON-1 or QGP-1 cells were treated with CPI203 as indicated, harvested at 48 h and analyzed by immunoblotting.

S1b. Increased G1 cell cycle arrest in BON-1 cells co-treated with CPI203 and rapamycin. BON-1 cells were treated with 50 nM CPI203, 100 nM rapamycin or in combination, harvested at 48 h, and analyzed for cell cycle profiles.

S1c. PARP immunoblot analysis in BON-1 cells treated with CPI203, rapamycin or in combination: BON-1 cells were treated with 50 nM CPI203, 100 nM rapamycin or in combination, harvested at 24 h and 72 h with corresponding control cells, and analyzed by immunoblotting.

Supplementary Figure S2 :

S2. CPI203 treatment slightly increased cell cycle arrest and slightly reduced cell proliferation in MYC-knockdown BON-1 cells. BON-1 cells were transfected with 25 nM siRNA oligos against *MYC* for 24 h, treated with CPI203 as indicated, evaluated for cell numbers (**a**) and analyzed for cell cycle profiles (**b**).
