# Supplementary figures and images for: The bromodomain and extra-terminal inhibitor CPI203 enhances the antiproliferative effects of rapamycin on human neuroendocrine tumors
Source: Cell Death Dis. 2014 Oct 9;5(10):e1450–. doi: 10.1038/cddis.2014.396 (PMC4237236; doi:10.1038/cddis.2014.396)

**a**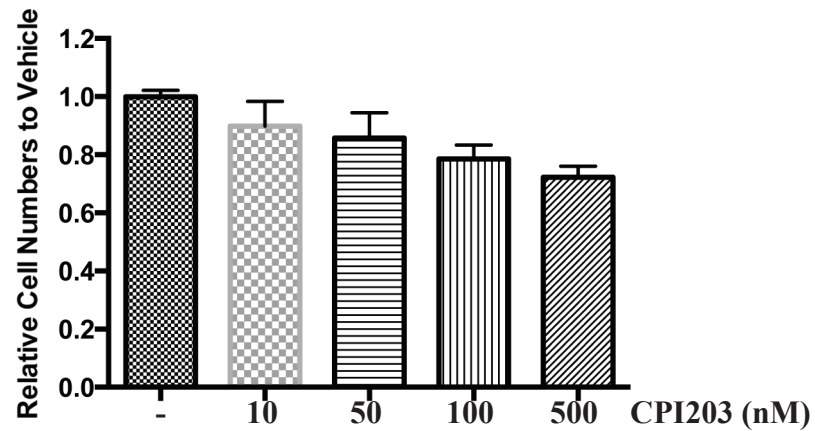**b**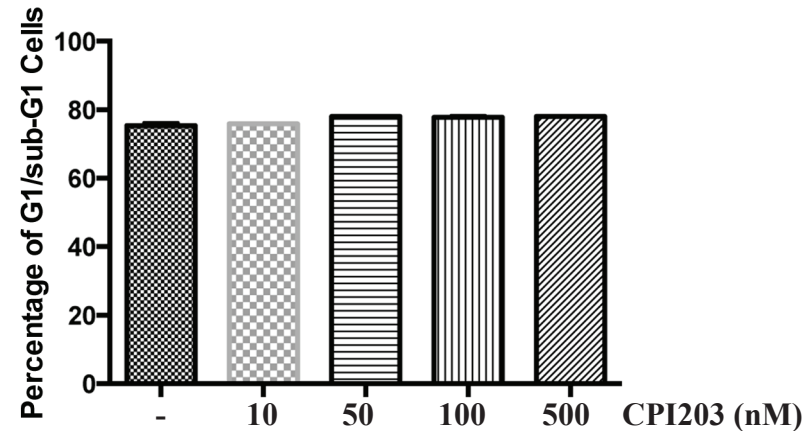

Supplement: Supplementary Figure S3 [file cddis2014396x3.pdf]
