# Supplementary material for: The bromodomain and extra-terminal inhibitor CPI203 enhances the antiproliferative effects of rapamycin on human neuroendocrine tumors
Source: Cell Death Dis. 2014 Oct 9;5(10):e1450–. doi: 10.1038/cddis.2014.396 (PMC4237236; doi:10.1038/cddis.2014.396)
Supplement: Supplementary Table Legends [file cddis2014396x6.doc]

**Supplementary Figure legends**

**Supplementary Table S1: Differentially down-regulated and up-regulated genes at 8 hour upon BET inhibitor CPI203 treatment:** BON-1 cells were treated with vehicle or 1 μM CPI203 and harvested at 8 h for global gene expression analysis as indicated in the methods.

**Supplementary Table S2 : Differentially down-regulated and up-regulated genes at 24 hour upon BET inhibitor CPI203 treatment:** BON-1 cells were treated with vehicle or 1 μM CPI203 and harvested at 24 h for global gene expression analysis as indicated in the methods.
