# Supplementary material for: The bromodomain and extra-terminal inhibitor CPI203 enhances the antiproliferative effects of rapamycin on human neuroendocrine tumors
Source: Cell Death Dis. 2014 Oct 9;5(10):e1450–. doi: 10.1038/cddis.2014.396 (PMC4237236; doi:10.1038/cddis.2014.396)
Supplement: Supplementary Figure S2 [file cddis2014396x2.pdf]

a

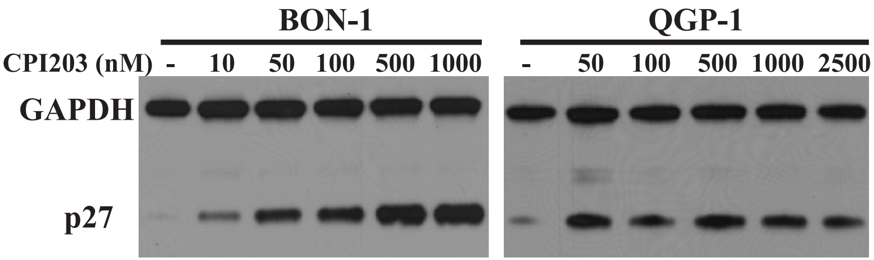

BON-1
QGP-1
CPI203 (nM) - 10 50 100 500 1000 - 50 100 500 1000 2500
GAPDH
p27

b

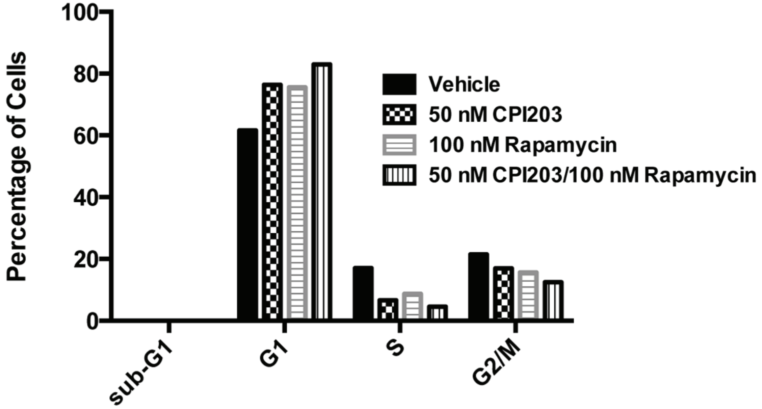

Percentage of Cells
100
80
60
40
20
0
sub-G1
G1
S
G2/M
Vehicle
50 nM CPI203
100 nM Rapamycin
50 nM CPI203/100 nM Rapamycin

c

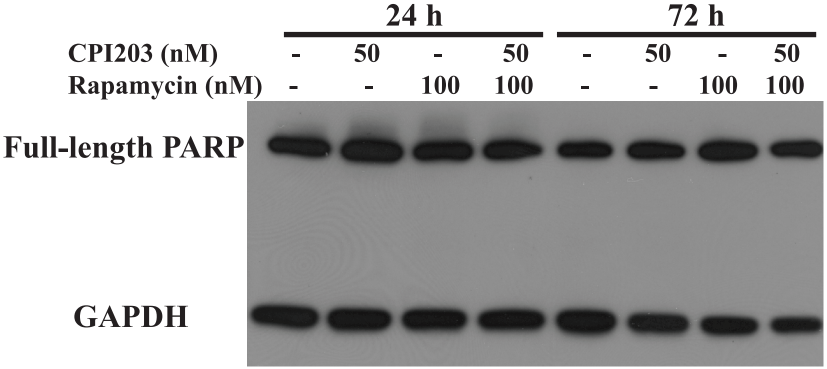

24 h
72 h
CPI203 (nM) - 50 - 50 - 50 - 50
Rapamycin (nM) - - 100 100 - - 100 100
Full-length PARP
GAPDH
